# Effect of the Microbiome on Intestinal Innate Immune Development in Early Life and the Potential Strategy of Early Intervention

**DOI:** 10.3389/fimmu.2022.936300

**Published:** 2022-07-19

**Authors:** Zhipeng Yang, Xiangchen Liu, Yanting Wu, Jian Peng, Hongkui Wei

**Affiliations:** ^1^ Department of Animal Nutrition and Feed Science, College of Animal Science and Technology, Huazhong Agricultural University, Wuhan, China; ^2^ The Cooperative Innovation Center for Sustainable Pig Production, Wuhan, China

**Keywords:** early life, microbiome colonization, intestine, innate immunity, probiotics, FMT

## Abstract

Early life is a vital period for mammals to be colonized with the microbiome, which profoundly influences the development of the intestinal immune function. For neonates to resist pathogen infection and avoid gastrointestinal illness, the intestinal innate immune system is critical. Thus, this review summarizes the development of the intestinal microbiome and the intestinal innate immune barrier, including the intestinal epithelium and immune cells from the fetal to the weaning period. Moreover, the impact of the intestinal microbiome on innate immune development and the two main way of early-life intervention including probiotics and fecal microbiota transplantation (FMT) also are discussed in this review. We hope to highlight the crosstalk between early microbial colonization and intestinal innate immunity development and offer some information for early intervention.

## 1 Introduction

Neonates are more susceptible to infections and other gastrointestinal diseases such as necrotizing enterocolitis (NEC) due to the immature immune system (1, 2). The ability to fight infections is dependent on innate immunity in early life (3); furthermore, the development of innate immunity is earlier than adaptive immunity and contributes to the maturation of adaptive immunity (4). The intestine is not only the digestive organ but also the body’s largest organ of immunity. More than half of the immune cells are stationed in the intestine, and 70% of immunoglobulin A (IgA) is produced in the intestine (5). Furthermore, as the closest part of the body to the outside environment, the intestine is the first line of defense. Thus, the development of innate immunity is important for neonates, while the specific mechanisms of immune modulation in early life also remain largely unknown.

Recently, emerging studies have unraveled that the development of the intestinal immune system is correlated with the abundance and diversity of the intestinal microbiome (6). After birth, the structure and diversity of the intestinal microbiome become more complex, and in parallel to the changes in the neonatal intestinal microbiome, immune cells in the intestine gradually mature. For example, higher production of cytokines at 36 months of age is closely related to colonization with *Bifidobacterium* at 1 week old (7).

The intestinal microbiome is easily influenced by dietary changes and the environment in early life (8); thus, neonatal intestinal microbial initial colonization is an important window of opportunity. Early intervention of intestinal microbiome colonization contributes to the development of the intestinal microbiome of neonates and has an impact on host immune development (9, 10). Probiotics and fecal microbiota transplantation (FMT) are the two effective ways to modulate intestinal microecology, promote the development of intestinal immunity, and maintain the intestine mucosa homeostasis, which has attracted a lot of interest in researchers as two main strategies of microbial intervention (11, 12).

Several reviews have summarized that microbial colonization shapes the immune system (13) and the effect of breast milk and solid food on the intestinal immunity (14), while this review provides an overview of the influence of microbial colonization on the development of intestinal innate immunity in early life especially focusing on the interaction between the specific bacteria and innate immune cells. Moreover, the effect of two main strategies, probiotics and FMT, of early intervention on intestinal innate immunity is also concluded. The goal of this review is to highlight the crosstalk between early microbial colonization and intestinal innate immunity development in early life and offer some references for early intervention by a microbial strategy.

## 2 Intestinal microbiome maturation in early life

### 2.1 Fetal Microbiome Composition

It has long been thought that the fetus is sterile and the establishment of the fetal microbiome begins after birth. The microbial community has been detected on human placental samples, cord blood, and meconium (15), while some researchers report that the experiment procedure, including sample collection and reservation, sequencing, and choosing of reagents are all different, and there is contamination during the whole trial. Furthermore, a lot of studies suggest that the human placenta has no microbiome by using different methods including 16S sequence, microbial cultivation, quantitative real-time PCR (qPCR), metagenomic next-generation sequencing (mNGS), and setting the negative control containing air and water (16–18). Whether the fetal microbiome exists remains a controversial issue. Here, we just present the microbiome found in the fetus.

Low diversity and limited bacteria have been detected in the mammal fetal intestine, skin, and lung by mNGS, 16S sequence, RNA-*in situ* hybridization (RNA-ISH), and scanning electron microscopy (SEM) (19, 20). A labeled *E. faecium* isolated from a healthy woman can be found in the amniotic fluid of the inoculated mice, which also indicated that the maternal microbiome can be transferred into the fetus (21). Phyla *Proteobacteria*, *Bacteroidetes*, *Actinobacteria*, and *Firmicutes* are enriched in the fetal intestine, especially *Enterobacteriaceae*, *Micrococcaceae*, and *Lactobacillus* (22–24). Additionally, bacterial metabolites such as short-chain fatty acids (SCFAs), deoxynojirimycin, mitomycin, and tobramycin exist in the fetal intestine, which also implies that there is a microbiome in the fetal intestine (23, 25).

### 2.2 Neonatal Microbiome Maturation

The microbiome of the newborn is similar to that of the mother and is closest to the vaginal microbiome. The intestinal microbiome in early life matures and evolves along with infant growth, and generally stabilizes and evolves adult-like after weaning, which has been proved *via* different ages of animal models, including mice (26) and pig (27), and infants (28).

Previous research supports the idea that oxygen is consumed by pioneer facultative anaerobic bacteria in the aerobic gut in early life, which then provides a niche for obligate anaerobes to colonize (29). On the first day after birth, facultative anaerobes from the *Proteobacteria* and *Firmicutes* (*Escherichia coli*, *Streptococcus*, *Enterococcus faecalis*, and *Enterobacter* sp.) are identified, as well as a low relative abundance of strict anaerobes from the *Bacteroidetes* and *Firmicutes* (*Bacteroides vulgatus*, *Parabacteroides*, and *Clostridium*) (30).

**Figure 1:**
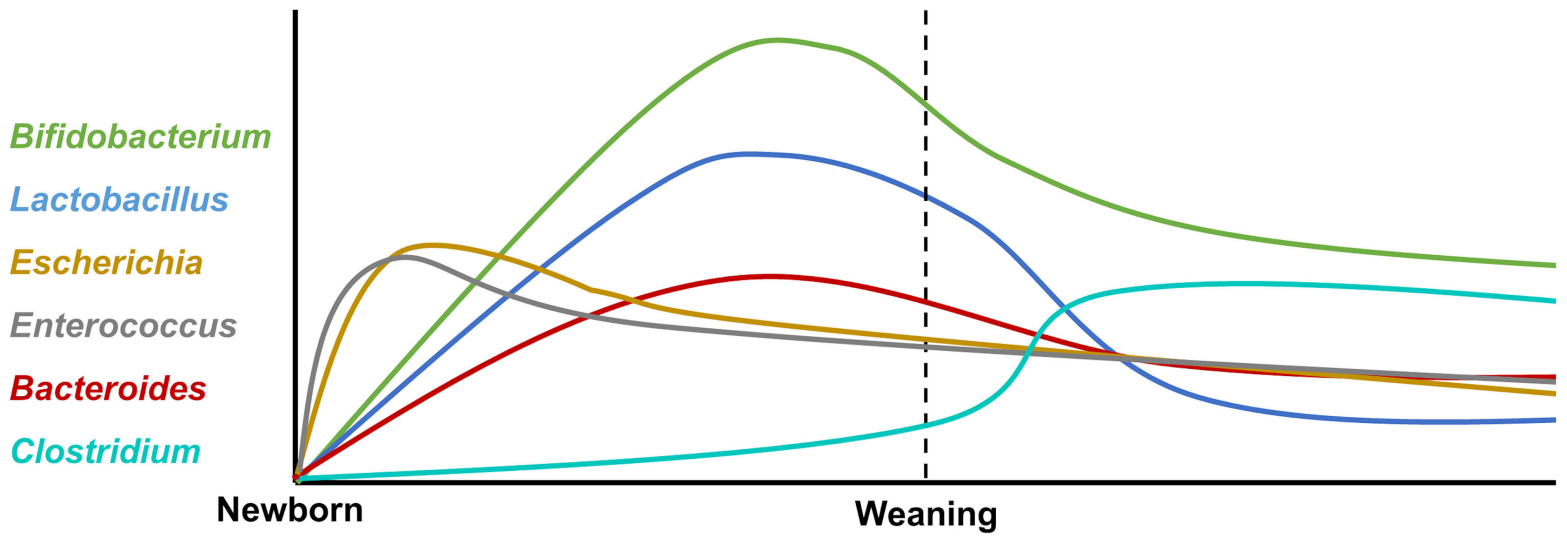
The variation of intestinal core microbiota after birth. The neonatal intestinal microbiota is mainly composed of *Bifidobacterium*, *Enterococcus*, *Lactobacillus*, *Escherichia*, *Bacteroides*, and *Clostridium*. After birth, facultative anaerobe bacteria *Escherichia* and *Enterococcus* increase rapidly and decrease gradually. The abundance of *Bifidobacterium*, *Lactobacillus*, and *Bacteroides* rises at the peak before weaning and then declines by degrees. *Clostridium* expands rapidly after weaning.

The neonates’ intestinal core microbiome is characterized by the predominance of *Bifidobacterium, Enterococcus, Lactobacillus, Escherichia, Bacteroides*, and *Clostridium* (31), and the variation after birth is shown in **Figure 1** *. Firmicutes* and *Bifidobacterium* dominate in the early developmental stages, which are characterized by an unstable community structure and low microbiome maturation. Along with infants growing up, *Bacteroides* and *Prevotella* gradually take the place of *Firmicutes* and *Bifidobacterium*, becoming high-level bacterial genera in infants (28). Normally, it is characterized by the predominance of such microorganisms as *Bifidobacterium longum* and *Bifidobacterium infantis*, *Bacteroidetes*, *Lactobacillus* sp., *Prevotella*, and *Atopobium*, which determine the direction of the immune response and the formation of tolerance in neonates (32).

After weaning, dietary changes from breast milk to solid food fixed with carbohydrates and fibers lead to major structural changes in the intestinal flora and microbiome diversity increasing. Furthermore, the intestinal microbiome structure tends to be relatively mature and stable; *Bifidobacterium*, *Lactobacillus*, and *Escherichia* decrease after weaning; and the intestinal flora are dominated by *Prevotella* (33). The mature microbiome contributes to the development of the innate immune response; for instance, the levels of cytokines related to immune cell proliferation and maturation were increased in fecal water of offspring (33). Furthermore, enterocyte proliferation is induced by intestinal microbiome maturation during early human life *via* microbial metabolites (34).

## 3 Intestinal innate immune system development

The intestinal epithelium and innate immune cells are the two main functional cells of the mammalian intestine innate immunity system. The intestinal epithelium consists of epithelial cells, Paneth cells (PCs) and goblet cells (GCs), and innate immune cells including macrophages, dendritic cells (DCs), natural killer (NK) cells, and innate lymphoid cells (ILCs) (35).

### 3.1 The Intestinal Epithelium

#### 3.1.1 Paneth Cells

PCs are housed in the crypt of the intestine and secrete antimicrobial peptides (AMPs), which are known to defend against pathogen infection (36, 37). Furthermore, PCs also play an essential role in intestinal homeostasis and microbiome colonization; for example, loss of PC function is implicated in microbiome dysbiosis and NEC in early life (38, 39). It has been found that *Cdk5rap3* (a gene that serves a vital role in intestinal PC development and maintenance) knockout resulted in early embryonic lethality, which indicated that embryonic PC function may determine the embryo fate (40).

PCs are detected in the small intestine at 13.5 weeks’ gestational age in fetus (41), then significantly increase to quantities similar to adults after 29 weeks’ gestational age (42), whereas it is worth noticing that PCs do not develop until 7–10 days after birth in mice (43). AMP gene expression such as cryptdins increases in an age-dependent manner from 3 to 21 days in rat (44, 45). After weaning, the number of PCs and the mRNA expression levels of AMPs including RegIIIβ, RegIIIγ, and Ang4 in the intestine were increased in mice (46, 47).

It has been demonstrated that PC number and functions are regulated by the intestinal microbiome, and germ-free (GF) mice have fewer intestinal PC numbers and RegIIIγ secretion compared with specific pathogen-free (SPF) mice (48). Early-life microbiome participates in the function and maturation of PCs, and the gene expression of PC products including defensins, matrilysin, and phospholipase A2 was significantly downregulated after antibiotic treatment in neonates (49). Furthermore, the number of PCs in a humanized microbiota gnotobiotic mouse model is significantly decreased compared with SPF mice, and the transportation of normal infant intestinal microbiome can rescue the low number of PCs and increase the AMP (Cathelicidin-5 and Lysozyme-1) mRNA expression level in the humanized microbiota gnotobiotic mouse model (50). On the other hand, the loss of a protective mucus layer leads to increased epithelial–bacterial interactions in mice and then results in the upregulation of RegIIIβ and RegIIIγ expression (46). RegIIIγ mRNA expression increased in the small intestine of conventionally raised mice throughout the weaning period, but not in GF animals, indicating that the intestinal microbiome is crucial for PC function during the weaning period (51).

#### 3.1.2 Goblet Cells

Intestinal GCs generate and secrete mucins, primarily mucin2 (MUC2), which are a major component of the mucus barrier, and serve as the first line of defense against pathogen infection and bacterial-derived product invasion (52, 53). Trefoil factor-2 (TFF2) deficiency in the neonatal small intestine allows the opportunistic neonatal pathogen *E. coli* K1 to permeate the weakened mucus layer in the distal small intestine and cause systemic infection (54). Intestinal GCs derive from intestinal stem cells (ISCs) that reside near the bottom of the intestinal crypt (55). It was recently discovered that GCs can form goblet cell-associated antigen passages (GAPs) and transfer luminal substances to antigen-presenting cells and DCs that underlie lamina propria (LP) and trigger adaptive immune responses (56).

In the human fetal small intestine, GCs first develop about 9–10 weeks of gestation, and both the stratified and simple columnar epithelium contains relatively undifferentiated oligomucous and mature GCs (57). Along with GCs development in the fetal intestine, MUC2 mRNA is detected as early as 9 weeks’ gestation, whereas MUC3 and MUC4, unlike MUC2, are expressed at a very early gestational age (as early as 6.5 weeks’ gestation) in humans, which expressed in the primitive gut endoderm before epithelial cytodifferentiation (58). In mice, shortly after villus emergence, around E16.5, the epithelium on the villi begins to undergo cytodifferentiation into GCs (59). After birth, the absolute number of GCs continues to expand, and the number of GCs in the intestines of 21-day-old piglets is almost double that of 1-day-old piglets (60).

The intestines of GF mice contain significantly fewer and smaller GCs compared with SPF mice (61). Furthermore, when GF mice are colonized with commensal bacteria, the size and number of GCs rapidly expand, and they release much Relm-β (62). Maturation of microbiome contributes to the function of GCs; functional maturation of GCs is more significant after exposure to adult microbiome than suckling microbiome, according to assessments of solute transporters and aquaporins (63). In a humanized microbiota gnotobiotic mouse model, the microbiome of an infant with poor weight gain has been shown to reduce the amount of GCs (50). The number of GCs was increased by transferring the microbiome from 15-day-old suckling conventional rats to the GF mice (63).

### 3.2 Immune Cells

#### 3.2.1 Dendritic Cells

DCs are bone marrow-derived antigen-presenting cells that are at the front line in maintaining intestinal integrity, and they comprise two major subsets: conventional (or classical) DCs (cDCs) and plasmacytoid DCs (pDCs). Intestinal DCs are found within the intestinal LP (64), Peyer patches (PPs) (65), and mesenteric lymph nodes (MLNs) (66). CD103^+^ DCs are key players in the innate immune system, and the relative paucity of CD103^+^ DCs in the neonatal intestine contributes to the high susceptibility to intestinal infection (67).

DCs are a bridge to connect innate immunity with adaptive immunity, which have been shown to stimulate T helper (Th) cells’ [Th1, Th17, and regulatory T cells (Tregs)] response in the intestine (68) and induce the activation and differentiation of naive B cells to yield plasma cells that produce commensal-specific IgA in the LP (69). Neonatal DCs are fully capable of priming CD8^+^ T cells during RV infection, and mice can elicit a robust antigen-specific CD8^+^ T-cell response, including the production of interferon gamma (IFN-γ) in early life (70). ResDCs induce Foxp3^+^ Tregs to provide a robust feedback mechanism for the maintenance of intestinal tolerance for the antigen of food and commensal bacteria in early life (71).

Bacterial signals during DCs differentiation have a profound impact on DCs function. DCs do not develop until day 16 in GF mice (72). DCs are highly microbiome dependent, and are disturbed following broad-spectrum antibiotic treatment, but can be restored by FMT, commensal *E. coli* and *Lactobacillus johnsonii* strains (73, 74). Moreover, MLN DCs from GF mice were less stimulatory for T cells than their counterparts from SPF mice (75).

#### 3.2.2 Innate Lymphoid Cells

ILCs are immune effector cells that contribute to host defense, metabolic homeostasis, and tissue repair (76). ILCs can be divided into three types: type 1 ILC includes IFN-γ producing ILC1, type 2 ILC includes interleukin-5 (IL-5) and IL-13 expressing ILC2, and type 3 ILC includes lymphoid tissue inducible cells (LTi) and IL-17 and IL-22 expressing ILC3 (77).

The early phase of ILC lineage commitment mainly occurred in the fetal liver and intestine. A considerable percentage of the lymphoid progenitor was found in the 8-week fetal intestine, which indicated that the intestine is an ILC-rich organ at fetal age, and ILC3 is a domain in the intestinal ILC family around week 12 (78). ILCs began working before the adaptive system has fully developed (79), early life is an important period for ILC maturation, and absolute ILC3 numbers increase dramatically from neonates to 14 days in mice (80). During the period of weaning, the proportion of phospho-signal transducer and activator of transcription 3 (pSTAT3)^+^ILC3 significantly increases, which indicates the activation of ILC3, and this phenomenon depends on IL-23 (79). After weaning, ILC3 activation is suppressed by the maturation of adaptive immune CD4^+^T cells, Treg, and Th17 cells (79).

It has been demonstrated that GF mice have been shown to have defective ILC3 development and lower production of IL-22, a key cytokine in epithelial responses to microorganisms in the small intestine (81, 82). Furthermore, prolonged transient neonatal antibiotic exposure suppresses IL-17A-producing ILC3 during the first 2 weeks of life, promoting bacterial translocation and increasing susceptibility to *K*. *pneumoniae*-induced sepsis, and recolonization with mature microbiome raises the level of IL-17A-producing ILC3 in mice, reducing their susceptibility to late-onset sepsis (LOS) (83).

#### 3.2.3 Natural Killer Cells

NK cells are recognized as the only cytotoxic population of ILCs. NK cells take part in protecting against pathogen infection including *Listeria monocytogenes*, *Salmonella*, *Citrobacter rodentium*, and *Yersinia enterocolitica* infections (84–86).

NK cells are detected in the intestinal epithelium and LP, which can be seen in fetal intestines (87). After birth, the number of NK cells increased dramatically from day 1 to day 3 (88). Early in life, a large proportion of this population was discovered (40% of IE lymphocytes in 9-day-old rats), with a distinct age-decreasing pattern (89).

CD103^+^ NK cells are the main congenital lymphocyte group in the small intestine of infants. Compared with adult intestinal NK cells, infant intestinal NK cells showed a stronger effect, with higher expression of the disintermediating embryonic protein, perforin, and granase B, and stronger degranulation ability (90).

The lower presence of NK cells has been detected in the intestine of GF mice (91). Microbiome impacts the epigenome of intestinal epithelial cells (IECs) and modifies interaction between IECs and NK cells through altering methylation-modifying enzyme activity (91). However, whether the microbiome can directly interact with NK cells to regulate NK cell activity and function remains to be determined.

#### 3.2.4 Macrophages

There is increasing evidence that macrophages play a crucial role in the maintenance of intestinal homeostasis and as key sentinels of the intestinal immune system. For example, macrophages enhance epithelial integrity by the production of prostaglandin E2 to stimulate ISC renewal (92). Moreover, macrophages maintain and expend Treg, Th17, and ILC3 through their secretion of cytokines such as IL-10, transforming growth factor beta (TGF-β), and IL-1β (93–95). Macrophages are first detected in the 11- to 12-week fetal intestine and the number rapidly increases from the 12-week to the 22-week period of gestation in human (96).

In comparison to conventionally housed controls, analysis of the colonic macrophage compartment in GF mice found markedly fewer monocyte–macrophage subsets in the colon (97). The microbiome also controls the homeostatic replenishment of monocyte-derived macrophages in the intestinal mucosa (97). In addition, genome-wide transcriptional profiling of macrophages from antibiotic-treated mice revealed a reduction in antiviral immunity genes (98). Furthermore, it has been demonstrated that early microbiome exposure stimulated tumor necrosis factor (TNF) production in spleen monocytes and macrophages of neonatal mice during the first 3 days of life (99) and the antibiotics caused an indirect or direct reduction in macrophage-like cells in the early life of chickens (100).

## 4 Microbiome and specific bacteria regulate the maturation of immunity in early life

In early life, the intestinal microbiome plays a crucial role in the development of the immune system. In detail, relative abundances of *Bacteroides* and *Prevotella* populations are highly correlated with responses to toll-like receptor-2 (TLR2) (101). Moreover, at 36 months of age, *Bifidobacterium* colonization is related to the greater production of IL-5, IL-6, IL-13, TNF, and IL-1. Gut colonization by *Enterococcus*, *Staphylococcus aureus*, or *Clostridium* in early childhood, on the other hand, was negatively associated with induced IL-13, IL-5, and TNF at 3 years of age (7). At 2 years of age, early *S. aureus* colonization was linked to a larger number of IL-4 and IL-10 generating cells (102).

Strong evidence has reported that early colonization of microbiome shapes the development of host immunity. Intestinal simulated epithelium exhibited a stronger epithelial barrier function and intestinal maturation when exposed to home-birth fecal supernatant, as well as a larger immune response (TLR4 route activation and pro-inflammatory cytokine release) (103). Furthermore, fungal colonization can lead to significant changes in intestinal bacterial composition in mice and independently affect systemic and intestinal innate and adaptive immunity in early life, and co-colonization of bacteria and fungi in the neonatal GF mice enhanced dextran sodium sulfate (DSS)-induced colonic inflammation in adulthood (104). As previously discussed, maternal microbial colonization during gestation increases ILC3 and mononuclear cell population size in offspring (105), further supporting the importance of the microbiome to the development of immunity in early life than in adults.

**Figure 2:**
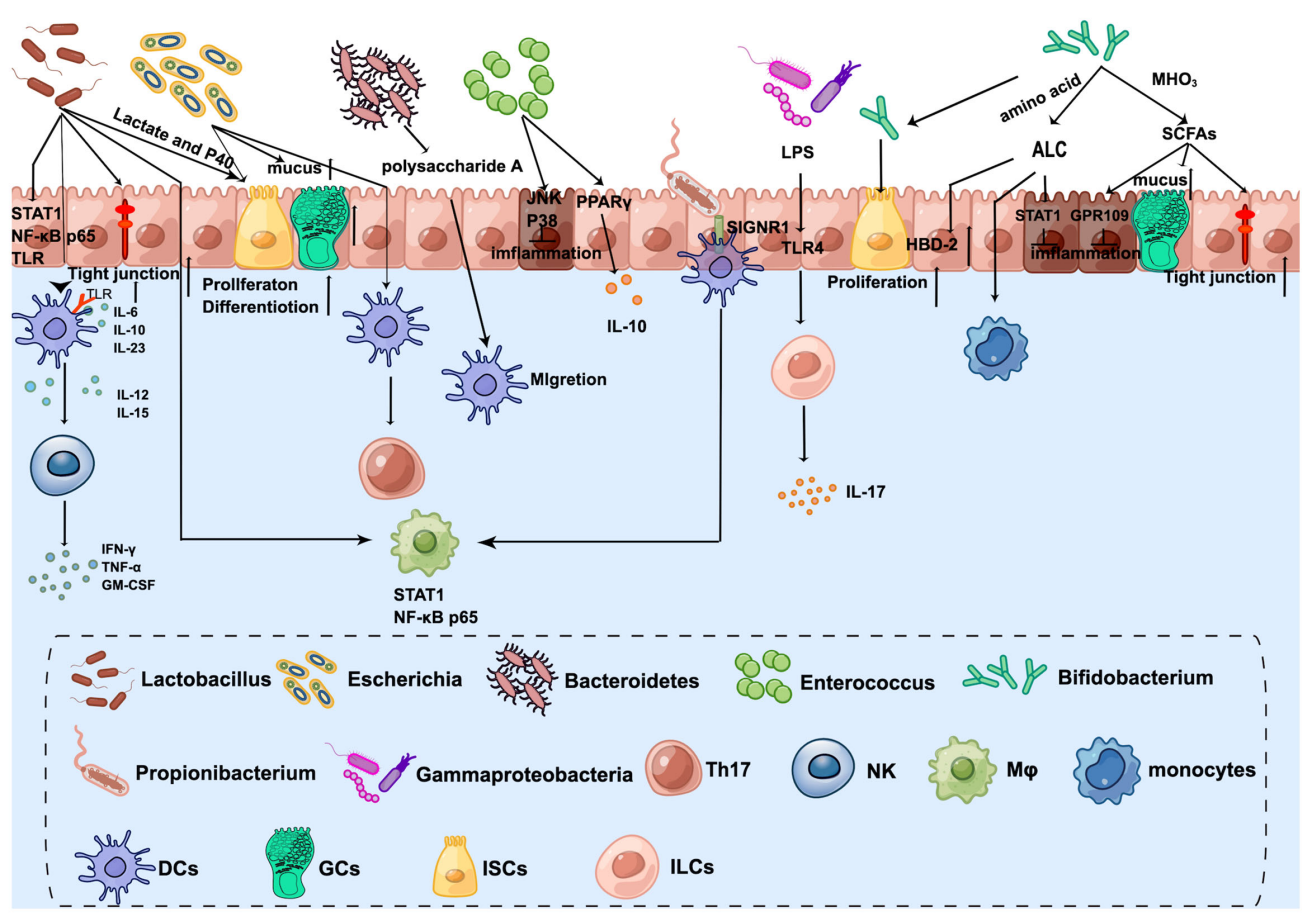
The crosstalk between microbiota and innate immune system in the neonatal intestine. *Lactobacillus* enhances DC differentiation and upregulates surface markers, which activates NK cells. Furthermore, STAT1 and NF-kB p65 nuclear translocation are influenced by *Lactobacillus* in both intestinal epithelial cells and underlying macrophages. *Lactobacillus* produced lactate and P40 can promote ISC-mediated epithelial development and tight junction formation. *Bifidobacterium* transfers amino acids into ALC, which activates monocytes and promotes the expression of beta-defensin2 of IECs. Additionally, ILA has anti-inflammatory effects on immature enterocytes through STAT1 pathways. *Bifidobacterium* releases SCFAs by metabolizing HMOs, which exert anti-inflammatory effects through GPR109A and increase tight junction and mucus gene expression. Furthermore, *Bifidobacterium* Tad pili stimulate the growth of the newborn mucosa. *Escherichia* increases the number of GCs and promotes mucus. The antigens of *E. coli* can be taken up by DCs and then present to CD4^+^ T cells to induce Th17. *Bacteroides*-produced Polysaccharide A mediates the migration of pDCs from the colon to the thymus to maintain PLZF^+^ lymphocyte homeostasis. *Enterococcus* attenuates proinflammatory cytokine secretions, especially IL-8 through JNK and p38 signaling pathways. Furthermore, *Enterococcus* promotes PPAR phosphorylation activity to activate the transcription of IL-10. *Propionibacterium* combines with SIGNR1 on the DCs to regulate macrophages. *Gammaproteobacteria* express cell-surface LPS, which is recognized by the host intestinal cells *via* TLR4 and then ILC3 produces the cytokine IL-17A. By Figdraw (www.figdraw.com).

As mentioned before, the infant intestinal core microbiome is characterized by the predominance of *Bifidobacterium, Enterococcus, Lactobacillus, Escherichia, Bacteroides*, and *Clostridium* (31). Early colonization of the microbiome after birth is helping to shape the innate immune system (**Figure 2**).

### 4.1 *Lactobacillus*

Milk-promoted bacteria such as *Lactobacillus* are signs of normal breast milk, and neonates are quickly colonized by *Lactobacillus* by suckling, which affects immune development in early life (106). The abundance of *Lactobacillus* in newborn mice as well as treatment with specific *Lactobacillus* probiotics (*L. murinus* and *L. johnsonii*) were able to resist *K. pneumoniae* colonization and LOS induced by *K. pneumoniae*, in contrast to eradicating the transmission of *Lactobacillus* by maternal antibiotic exposure exacerbated disease (107). Moreover, the enrichment of *Lactobacillus* is associated with low NEC scores in the NEC rat model; in detail, *L. murinus* isolated from neonatal rats reduces molecular markers and the intestinal barrier damage of NEC (108).

Infant’s feces-derived *Lactobacillus* appears to be a promising modulator of innate immune response, which can enhance DC differentiation including promoting the secretion of IL-6, IL-10, IL-12, IL-15, and IL-23 and upregulate surface markers (HLA-DR, CD86, CD83, and CCR7) in DCs (109), which activates NK cells and promotes the synthesis and secretion of INF-γ, TNF-α, and granulocyte-macrophage colony-stimulating factor (GM-CSF); the presence of *Lactobacillus* switches the immune system towards a Th1 response in early life (110, 111). Another type of *Lactobacillus*, isolated from exclusively breast-feeding infant feces, activates TLR signaling of DCs and reduces *Salmonella*-induced inflammation (112). Moreover, a co-culture model of the undeveloped small intestine and underlying macrophages showed that STAT1 and nuclear factor kappa B (NF-kB) p65 nuclear translocation are influenced by *Lactobacillus* in both IECs and underlying macrophages, which offers new insights into the potential routes by which *Lactobacillus* primes the early immune system to fight pathogens (113).

Though a lot of evidence shows that *Lactobacillus* modulates the innate immune system in early life, knowledge about the mechanisms underlying regulating innate immune development by *Lactobacillus* is limited. P40, a *Lactobacillus rhamnosus* GG-derived protein, enhances functional maturation of the intestine in early life including IEC proliferation, differentiation, and tight junction formation by activation of epidermal growth factor receptor (114). Furthermore, *Lactobacillus*’ SpaC pilus was found to adhere to the fetal gut and to modulate IECs’ innate immune gene TLR-related gene expression and attenuate pathogen-induced TNF-α mRNA expression (115). Lactate, mainly produced by *Lactobacillus*, is the most abundant metabolite in the intestine during the first 6 months of life (116), and is a key factor in promoting ISC-mediated epithelial development. In detail, lactate directly stimulates PCs, and intestinal stromal cells through Gpr81 activation of the Wnt3–β-catenin pathway and plays an important role in promoting the proliferation of Lgr5^+^ ISCs (117). Furthermore, the effect of lactate on the proliferation of ISCs in newborn mice was greater than that in adult mice (117).

### 4.2 *Bifidobacterium*

*Bifidobacterium* is among the earliest and most dominant colonizers of the neonatal gut and is closely related to intestinal development. Colonization of the infant intestine with *Bifidobacterium* is influenced by human milk oligosaccharides (HMOs) from breastmilk. HMOs stimulate the growth of *Bifidobacterium* in the intestine, which offers a nutrient source for the infant intestinal microbiome, especially *Bifidobacterium* species that encode HMO-utilizing genes (118). Lack of *Bifidobacterium* and its breast milk oligosaccharide utilization genes is associated with systemic inflammation and immune imbalance in early life (119).

Aromatic lactic acids (ALAs) include indolelactic acid (ILA), phenyllactic acid (PLA), and 4-hydroxyphenyllactic acid (4-OH-PLA), which is a metabolite produced from the aromatic amino acids tryptophan, phenylalanine, and tyrosine by *Bifidobacterium* utilizing aromatic lactate dehydrogenase. ALAs are associated with the activation of aryl hydrocarbon receptors (AhRs) and regulate intestinal homeostasis. It has been demonstrated that ILA acts as an agonist for both the AhR and the hydroxycarboxylic acid receptor 3 (HCA3) and affects *ex vivo* immunological responses of human CD4^+^ T cells and monocytes in a dose-dependent way (120). Furthermore, ILA activates AhR and attenuates damaging pro-inflammatory responses after IL-1β stimulus and increases the expression of pBD-2 in IECs (121, 122). Transcriptomic analysis was used to demonstrate that immature enterocytes are protected by ILA in a variety of ways, including anti-inflammatory and antiviral activities. STAT1 pathways are critical for IL-1β-induced inflammatory response, and STAT1 gene transcription by ILA is essential for maintaining healthy intestinal development (123).

SCFAs are released by *Bifidobacterium* species metabolism of HMOs using intracellular glycosyl hydrolases and are by-products that can be used by the host (124). The SCFA concentration in the intestine sharply increases after birth (125). SCFAs are known to have anti-inflammatory properties in mature enterocytes and immune cells. Their role in the innate immunity of the developing intestine is not well understood. A study suggested that an immature intestinal mechanism for reducing inflammation differs from that of the mature intestine. SCFAs activate G-protein-coupled receptor 109A (GPR109A) in immature enterocytes in response to IL-1 stimulation, then inhibit IL-1-induced activation of HDAC3 and HDAC5 genes to reduce inflammatory responses (126). Furthermore, when exposed to an IL-1β inflammatory stimulus, acetate, propionate, and butyrate were all anti-inflammatory. Butyrate induces an anti-IL-1 response by increasing tight junction and mucus gene expression (127). Sodium butyrate administration significantly reduced the expression of HMGB1 and pro-inflammatory cytokines and decreased intestinal inflammation in the NEC model (128).

*Bifidobacterium* Tad pili may play a role in the early maturation of the naive gut by producing a unique scaffold of extracellular protein structures that stimulate the growth of the newborn mucosa (129). Furthermore, in human embryonic colon epithelial cells, *Bifidobacterium* regulates intestinal development by stimulating cell proliferation and accelerating the maturation of the internal barrier *via* the Wnt/β-catenin and PI3K/Akt/mTOR signaling pathways (130).

### 4.3 *Escherichia*

Aerobic conditions initially help in the rapid colonization of facultative anaerobic species in the neonatal intestine, such as *E. coli*. Colonization in the early stages with *E. coli* causes epithelial remodeling in the colon, changing the epithelium’s structure as well as the mucus layer. For example, proliferative cell markers (Ki67, proliferating cell nuclear antigen, phospho-histone H3, and cyclin A) were higher in rats non-colonized with *E. coli* than in GF, and following *E. coli* inoculation, epithelial MUC2 and mucopolysaccharide levels decreased rapidly, implying that these components are released to join the mucus layer, which doubled in thickness after inoculation (131). Furthermore, the intestinal microbiome composition of newborns was closely related to that of their mothers. Intestine epithelial cell differentiation is regulated by transmitting *E. coli* from the mother to the neonates, which increases the numbers of GCs (132). It has been demonstrated that *E. coli* EC 25 isolated from neonatal rats protects neonatal rats from NEC (133).

During days 0–10 of life, immune system exposure to luminal bacteria is limited by growth factors. Then, bacterial antigens are delivered to the LP by GAPs and captured as well as processed by lamina propria DCs (134). The antigens of *E. coli* can be taken up by DCs and then present to CD4^+^ T cells to induce Th17 (135).

The oxygen-rich environment in the intestinal tract during the neonatal period is the main factor affecting the colonization of bacteria and driving the excessive growth of *K. pneumoniae.* The oxygen content in the intestinal tract decreases gradually with the development, forming mature bacteria dominated by anaerobic microorganisms that can resist *K. pneumoniae* (107). In addition, commensal *Enterobacteriaceae* contribute to colonization resistance by competing for oxygen with *S. enteritidis*, which is necessary for pathogen expansion (136).

### 4.4 *Bacteroides*

*Bacteroides* species are found in varying amounts in the baby’s intestinal microbiome, but by the first year of life, they are always present (137). Indeed, Polysaccharide A, produced by *Bacteroides fragilis*, promotes PLZF^+^ lymphocyte homeostasis in the thymus through TLR2 signaling, while the colony mediates the migration of pDCs from the colon to the thymus to maintain PLZF^+^ lymphocyte homeostasis, and intervening intestinal flora in early life by antibiotic affects the distribution of PLZF^+^ cells in the adult thymus and then increases susceptibility to DSS-induced colitis (124).

### 4.5 *Enterococcus*

*E. faecalis* dominates more than 60% of the total LAB in the stool of healthy newborns and represents a more effective competitor than the rest in initiating colonization of a newborn (138). *In vivo* and *in vitro*, *E. faecalis* attenuates inflammation by suppressing proinflammatory cytokine secretions, especially IL-8 (139). Furthermore, peroxisome proliferator-activated receptor (PPAR) activity can be altered by *E. faecalis* through phosphorylation, resulting in increased DNA binding and transcriptional activation of IL-10 (134).

### 4.6 Others

Human breast milk-fed preterm infants are colonized by commensal *Propionibacterium* with high abundance, and the P.UF1 bacterium combines with SIGNR1 on the DCs to regulate macrophage in the intestine, which contributes to protect against pathogen infection and mitigate induced NEC-like injury (140).

It has been demonstrated that there is a lower level of *Akkermansia muciniphila* in oversized infants, although there was no direct evidence showing the crosstalk between *Akkermansia* and intestinal immunity in newborns (141, 142). A study on enterocytes implies that *A. muciniphila* may alleviate obesity by strengthening the epithelial barrier in infants (143).

The bacteria that colonize a newborn’s gut in the first few days after birth are all from the class Gammaproteobacteria, Gram-negative bacteria that express cell-surface LPS, which is recognized by the host intestinal cells *via* TLR4. ILC3 produces the cytokine IL-17A as a result of this signaling sequence. Upregulation of IL-17A in the gut causes G-CSF to recruit neutrophils from the bone marrow into the bloodstream, increasing circulating neutrophils that effectively remove blood-borne infections like *E. coli* K1 (144).

## 5 Ways to early intervene

Early life is a critical period for the development of the intestinal innate immune system, and increased exposure to environmental microorganisms during this period may improve immune function by regulating the symbiotic microbiome. For example, microbial exposure early in life influences immune maturation; it may reduce the risk of certain conditions later in life, such as colitis, by enabling the intestinal microbiome to develop properly during the early stages of immune development (9, 10).

### 5.1 Probiotics

Probiotics are defined as live microorganisms that produce health benefits and have been investigated by humans for centuries. Strong evidence supports the contribution of probiotics to maintaining intestinal mucosa homeostasis and the development of immunity in early life. Furthermore, the ability to resist DSS- and *C. rodentium*-induced intestinal injury and intestinal tumor formation in adulthood can be enhanced by early-life intervention (114, 145, 146). Hence, the effect of probiotics on early life has attracted many researchers.

The impact of probiotics on intestinal innate immune development in early life is summarized in **Table 1**. As mentioned above, *Lactobacillus* and *Bifidobacterium* are milk-promoted bacteria in the neonatal intestine; thus, one of the early probiotics intervention strategies is to promote the colonization of these bacteria. Early administration of *Bifidobacterium* and *Lactobacillus* mainly enhanced the function of epithelial cells. The numbers and function of PCs and GCs are increased by supplementation with *Bifidobacterium* and *Lactobacillus* including the expression of TFF3, Lysozyme-1, β-defensin-1 (pBD-1), and pBD-2, and the secretion of MUC2, MUC3, and MUC4 (150, 154–156). The underlying mechanism may be through the bacteria-derived lactate (117). Furthermore, bacteria pilus was found to not only activate the TLR (115), and *Bifidobacterium* and *Lactobacillus* supplements in early life but also enhance the expression of TLR such as TLR2, TLR4, and TLR9. Moreover, early administration of *Bifidobacterium* enhances the secretion of TNF-α, IL-12, IL-10, IFN-γ, IL-6, CX3CL1, and IL-22. In addition, colonization of *B. longum* and *Lactobacillus reuteri* enhances the proliferation and the ability of antigen-presenting cells, which promote the development of adaptive immunity (151, 157, 159). It is worth noting that the co-supplementation of *Bacillus subtilis* and *Lactobacillus salivarius* stimulates a more significant mucosal immunity than the supplementation of each bacteria alone (149).

**Table 1:** The effect of probiotics on the intestinal innate immunity.

Probiotic	Object	Does (CFU)	Treatment period	Effect	Ref.
*Bifidobacterium breve M-16V*	Rat	5 × 10^7^–2 × 10^8^	D6–D18	TLR4^+^ cells↑	(147)
*Bifidobacterium pseudocatenulatum CECT 7765*	Mice	1 × 10^8^	D2–D21	TNF-α↑	(148)
*Bifidobacterium. subtilis RJGP16*	Pig	5 × 10^9^, 10^10^, 1.5 × 10^10^	D1, D7, D11	IL-6, TLR2↑	(149)
*Bifidobacterium bifidum G9-1*	Mice	3 × 10^7^	D1–D7	MUC2, MUC3, MUC4↑ TGF-β1, TFF3 ↑ Acidic mucin↑	(150)
*Bifidobacterium longum*	Rat	1 × 10^10^	D1–D21	DCs↑ IL-12, IL-10, IFN-γ↑	(151)
*Bifidobacterium longum* subsp. *infantis*	Rat	5 × 10^6^	D4–D7	TFF3↑	(152)
*Lactobacillus delbrueckii*	Pig	5 × 10^9^	D1, D3, D7, D14	IL-6, CX3CL1↑	(153)
*Lactobacillus reuteri*	Pig	10^9^	D3–D8	GCs↑ Tcf4↑ MUC2, Lysozyme-1, pBD-1, IL-22↑ The area of PP↑	(154)
*Lactobacillus salivarius*	Pig	5 × 10^9^, 10^10^, 1.5 × 10^10^	D1, D7, D11	TLR2, pBD-2 ↑	(149)
*Lactobacillus salivarius*	Pig	5 × 10^9^, 10^10^, 1.5 × 10^10^	D1, D7, D11	pBD-2↑	(155)
*Lactobacillus casei*	Rabbit	5–6 × 10^8^	D5–D13	PCs ↑ TLR9, Defen, lysozyme↑	(156)
*Lactobacillus reuteri DSM 17938*	Mice	10^6^	D5–D9	DCs↑	(157)
*Lactobacillus reuteri DSM 17938*	Pig	10^6^	D1–D3	IL-10↑ IL-6, TNF-α, TLR4, and NF-κB↓	(158)
*Lactobacillus reuteri DSM 17938*	Rat	5–10 × 10^9^	D1–D4	DCs↑ IL-1β↑	(159)
*Bifidobacterium subtilis RJGP16* *Lactobacillus salivarius*	Pig	5 × 10^9^, 10^10^, 1.5 × 10^10^	D1, D7, D11	IL-6, TLR2, pBD-2↑	(149)
*Lactobacillus delbrueckii*	Pig	5–20 × 10^9^	D1, 3, 7, 14	TLR2, TLR4↑ DCs produced TNF-α, IL-12↑	(160)
*Lactobacillus acidophilus*	Mice	1.25 × 10^8^	D14–D28	DCs↑	(161)
*Lactobacillus rhamnosus GG*	Mice	1 × 10^7^	D1–D5	GCs↑	(162)
*Lactobacillus* salivarius B1	Pig	5, 10, 15, 20 × 10^9^	D1, D7, D11, D26	pBD-2↑	(155)
*Lactobacillus paracasei*	Mice	10^9^	D1–D14	MUC2↑ Ki67↑ ZO-1↑	(163)
*Lactobacillus reuteri*	Chick	10^8^	D3–D7	GCs↑ MUC2↑ lysozyme↑	(164)
*Enterococcus faecium EF1*	Pig	1–1.2 × 10^9^	D1, D3, D5	TGF-β and TNF-α↑ TLR2 and TLR9↑	(165)
*Enterococcus faecium EF1*	Pig	6 × 10^8^	D1, D3, D5	IL-10 and TGF-β↑	(166)
*Bacillus subtilis RZ001*	Mice	1 × 10^7^	D2–D15	GCs↑ MUC2↑	(167)
*Escherichia coli* Nissle 1917	Pig	10^5^	D6–D20	pDCs↑ IL-12↑	(168)
*Lactobacillus casei* *Enterococcus faecalis*	Pig	1–4 × 10^9^	D1, D7, D14, D21	TGF-β↑ TNF-α↓	(169)
*Saccharomyces boulardii*	Pig	1 × 10^10^	D4–D24	pBD2, pBD3, pBD114, pBD129↑ MUC1↑	(170)

↑, up regulation; ↓, down regulation.

Moreover, other probiotics such as *B. subtilis* RZ001 and *Enterococcus faecium* EF1 also exert a regular function in innate immunity. Oral administration of *B. subtilis* RZ001 in suckling mice increases the number of PCs and the expression of MUC2 by promoting the proliferation of ISCs through Wnt signaling (167), while it is worth noticing that *B. subtilis* increased the relative abundance of lactate-producing bacteria, which may be the main reason for its effect. *E. faecium* EF1 supplementation in suckling pigs enhanced the innate immunity-related mRNA expression of toll-like receptors TLR2 and TLR9 and cytokines TGF-β and TNF-α (165).

It has been demonstrated that early-life intervention by probiotics contributes to protecting against pathogen infection (**Table 2**). The defense against different kinds of invasive *E. coli* including K88, K1, and F4 was enhanced by supplanting probiotics by promoting the maturation of neonatal intestinal immune response and enhancing the mucosal barrier. For instance, co-administration of *Lactobacillus bulgaricus*, *Bifidobacterium*, and *Streptococcus thermophilus* promotes the production of mucin, ZO-1, and Ki67 in intestinal mucosa to prevent neonatal *E. coli* K1 translocation (176). Furthermore, LGG reverses the intestinal injury of neonatal mice against *Salmonella* challenge by increasing MUC2 and tight junction gene expression (172), and rotavirus infection can also be prevented by the early intervention of LGG (173).

**Table 2:** The effect of probiotics on intestinal pathogen infection.

Probiotic	Object	Does (CFU)	Treatment period	Pathogen	Ref.
*Lactobacillus plantarum*	Pig	Unknown	D4–D14	*E. coli* K88	(171)
*Lactobacillus rhamnosus GG*	Mice	Unknown	D4	*Salmonella typhimurium*	(172)
*Lactobacillus rhamnosus GG*	Rat	1.5 × 10^8^	D2–D7	*Rotavirus*	(173)
*Enterococcus faecium* NCIMB 10415	Pig	3 × 10^9^	D2–D8	*E. coli* K88	(174)
*Lactobacillus salivarius*	Pig	2 × 10^9^	D1–D3	*E. coli* F4	(175)
*Lactobacillus bulgaricus, Bifidobacterium, Streptococcus thermophilus*	Rat	1 × 10^9^	D2–D5	*E. coli* K1	(176)
*Lactobacillus* GG	Rat	1–2 × 10^5^	D3–D4	*E. coli*	(177)

A recent human intestinal mucosal transcriptome study assessing the effect of the administration of different probiotics has shown that changes in gene expression are more dependent on the individual than on the probiotic strain used (178); thus, the co-administration of probiotics is a good strategy to replace the single bacteria. Moreover, gene engineering strains are another useful strategy for early intervention. For example, a recombinant strain of *L. reuteri* secreting biologically active Lfampin (LR-LFCA) was developed; LR-LFCA enhances immune response and is beneficial for the intestinal health of neonatal piglets through improving intestinal barrier function and modulating the composition of gut (179).

Probiotics early intervention is increasingly being used in the clinic; a meta-analysis that included 10,812 infants demonstrated that early intervention with probiotics including *Bifidobacterium* spp., *Lactobacillus* spp., *Saccharomyces* spp., and *Streptococcus* spp. reduced the risk of NEC, late-onset invasive infection, and mortality in very-preterm or very-low-birth-weight infants (180). Moreover, the intervention that combined *Bifidobacterium* and *Lacticaseibacillus* accelerated microbial maturation such as α-diversity, stability, and interconnectivity and reduced the inflammatory response in extremely preterm infants (181). Probiotics are an effective strategy to prevent gastrointestinal disease in infants, and research showed that 14% of intensive care units administered probiotics to low-birth-weight premature babies between May and September 2015 in the USA (182). Clinical trials on probiotics aim to determine their safety and effectiveness, although their effect has been investigated in experimental animals such as mice, rats, and pigs.

### 5.2 FMT

In recent years, as a strategy of intestinal microecological regulation apart from probiotics, FMT has become popular in recent years. FMT was mainly used to reverse the disorder gut microbiome ecosystem in *Clostridioides difficile* infection and inflammatory bowel disease (IBD) adulthood to achieve the therapeutic goal (183–185). In neonates, FMT not only promoted the diversity and composition of the intestine but also regulated the landscape of microbiota metabolites (186).

As shown in **Table 3**, there is little research on the effect of FMT in the development of intestinal innate immunity at present. FMT in the neonates promoted the development of GCs and the secretion of MUC2 (153, 187, 188, 192). Moreover, the expression of pBD-2, pBD-3, PMAP-23, and PAMP-37 by PCs was raised by FMT in the suckling piglet (187, 189). In addition, the expression of TLR such as TLR2 and TLR4, and the innate immune cytokines including IL-2, IL-6, IL-23, IL-17, IL-22, and INF-γ was enhanced after FMT (188–190), whereas the development and function of other types of innate immune cells including DCs, ILCs, NK, and macrophages were not been reported.

**Table 3:** The effect of FMT on the intestinal innate immunity.

Donor	Dose (g/day)	Receptor	Treatment period	Effect	Ref.
Sow	0.075	Pig	D1–D11	GCs↑ MUC2↑ pBD2↑ TLR2, TLR4↑	(187)
Sow	0.05	Pig	D3–D7	TLR2, TLR-↑ MUC2↑	(188)
Sow	D1–D2: 0.3 D3–D10: 0.6	Pig	D1–D10	IL-2, IFN-γ, IL-6↑	(189)
Sow	0.05	Pig	D1–D3	IL-23, IL-17, IL-22, INF-γ↑	(190)
Sow	0.05	Pig	D1–D3	MUC2↑	(191)
10-day-old piglet	0.05	Pig	D1–D2	GCs↑	(192)
Sow	0.2	Pig	D3–D7	MUC2↑ TLR2, TLR4↑	(188)
Sow	0.3	Pig	D4–D6	IL-10, TGF-β1↑	(193)
Adult chicken	0.025	Chick	D1	NK cells↑	(194)

↑, up regulation.

NEC is in part due to excessive inflammation in the immature intestine by colonizing bacteria because of an immature innate immune response (127). Evidence of a potential impact of FMT on reducing the risk of newborns with NEC has been shown. FMT before or after NEC induction can decrease the mucosa damage, inflammatory response (level of TNF-α, IL-1β, and IL-6), and oxidative damage [myeloperoxidase (MPO) activity] (195). Fecal filtrate transplantation (FFT) also prevented NEC in preterm neonates, and the orogastric administration was better than fecal filtrate transfer rectally (196).

Several studies have been reported on the FMT influence of pathogen infection. Compared with suckling pigs without FMT, FMT alleviated enterotoxigenic *E. coli* (ETEC) K88 infection caused by mucosa injury including the decrease in the number of GCs and the production of MUC2 (197). Lipopolysaccharide (LPS) is the major component of the outer membrane of Gram-negative bacteria, such as PAMPs, and LPS induces the damage of the mucosa integrity. FMT promoted the microbiome-derived indole acetic acid (IAA) from tryptophan, which maintains the intestinal barrier by the induction of IL-22, thus reducing susceptibility to LPS-induced destruction of epithelial integrity and severe inflammatory responses (186).

Of note, colonization of microbiome from different intestinal segments was region-specific, and compared with FMT, whole-intestinal microbiota transplantation (WIMT) is more conducive to the transplantation of the donor intestinal microbiome and its function into the recipient small intestine (198). However, it is worth noticing that not just any microbiome from any animal can promote immune system development and maturation in mice; the intestine immune system only depends on colonizing with a host-specific microbiome. For example, the number of GF mice’s T cells could only be reconstructed by mouse microbiome instead of human or rat microbiome (199).

## 6 Conclusion and perspectives

Intestinal innate immunity is one of the first defense lines of the host. The intestinal microbiome plays a crucial role in the development of the host’s immune system in early life. Here, we summarize how the intestine microbiome directs the development of the intestinal innate immune system composed of the epithelium and immune cells from early life. In neonates, the intestinal innate immunity is shaped by the milk-derived bacteria *Lactobacillus* and *Bifidobacterium*, and other commensal bacteria including *Escherichia*, *Bacteroides*, and *Enterococcus*. Early intervention by probiotics and FMT are two effective ways to modulate intestinal microecology, promote the development of intestinal immunity, and maintain intestinal mucosa homeostasis. Nevertheless, the impact of probiotics and FMT on other immune cells such as ILCs is not yet researched. Furthermore, a single probiotic or FMT may not be the best strategy for early intervention, and FMT carries the risk of facing an infectious disease because of the harmful strains (200). More focused efforts to uncover a combination of multiple bacteria can be developed to enhance neonatal intestinal health. For instance, the immune system was enhanced by colonizing the synthetic community (SYN) in the cecum, which is composed of 9 different commensal bacteria from chicken (201). Thus, the research on core bacteria and the combination of multiple bacteria is the trend for early intervention in the future. Moreover, clinical tools including sequence style and bioinformatics to detect and predict the intestinal microbiome in infants are worthy of further research. For example, bioinformatics accelerates the pharmaceutical industry (202). qPCR was used to detect the specific bacteria that correlated to disease or intestinal health in the clinic (203). Moreover, the occurrence of NGS and metagenomic sequencing of the microbiome helps us investigate the microbial composition deeply (204), and the different methods of analysis such as *via* a co-occurrence network, functional annotation, and random forest (181, 205) help us predict the maturation of the intestinal microbiome and exploit new probiotics for infants.

## Author Contributions

ZY designed and wrote the manuscript. XL completed the table. YW drew the figure. HW and JP designed the manuscript and provided funding and modified the manuscript. All authors contributed to the article and approved the submitted version.

## Funding

This research was supported by Hubei Province Science and Technology Innovation Major Project (2021BBA083) and China Agriculture Research System of MOF and MARA.

## Conflict of Interest

The authors declare that the research was conducted in the absence of any commercial or financial relationships that could be construed as a potential conflict of interest.

## Publisher’s Note

All claims expressed in this article are solely those of the authors and do not necessarily represent those of their affiliated organizations, or those of the publisher, the editors and the reviewers. Any product that may be evaluated in this article, or claim that may be made by its manufacturer, is not guaranteed or endorsed by the publisher.
